# Monte Carlo simulation of the effect of melanin concentration on light–tissue interactions for transmittance pulse oximetry measurement

**DOI:** 10.1117/1.JBO.29.S3.S33305

**Published:** 2024-08-13

**Authors:** Raghda Al-Halawani, Meha Qassem, Panicos A. Kyriacou

**Affiliations:** City, University of London, Research Centre for Biomedical Engineering, London, United Kingdom

**Keywords:** Monte Carlo simulation, skin pigmentation, melanin, calibration, pulse oximetry

## Abstract

**Significance:**

Questions about the accuracy of pulse oximeters in measuring arterial oxygen saturation (${\mathrm{SpO}}_{2}$) in individuals with darker skin pigmentation have resurfaced since the COVID-19 pandemic. This requires investigation to improve patient safety, clinical decision making, and research.

**Aim:**

We aim to use computational modeling to identify the potential causes of inaccuracy in ${\mathrm{SpO}}_{2}$ measurement in individuals with dark skin and suggest practical solutions to minimize bias.

**Approach:**

An *in silico* model of the human finger was developed to explore how changing melanin concentration and arterial oxygen saturation (${\mathrm{SaO}}_{2}$) affect pulse oximeter calibration algorithms using the Monte Carlo (MC) technique. The model generates calibration curves for Fitzpatrick skin types I, IV, and VI and an ${\mathrm{SaO}}_{2}$ range between 70% and 100% in transmittance mode. ${\mathrm{SpO}}_{2}$ was derived by inputting the computed ratio of ratios for light and dark skin into a widely used calibration algorithm equation to calculate bias (${\mathrm{SpO}}_{2}-{\mathrm{SaO}}_{2}$). These were validated against an experimental study to suggest the validity of the Monte Carlo model. Further work included applying different multiplication factors to adjust the moderate and dark skin calibration curves relative to light skin.

**Results:**

Moderate and dark skin calibration curve equations were different from light skin, suggesting that a single algorithm may not be suitable for all skin types due to the varying behavior of light in different epidermal melanin concentrations, especially at 660 nm. The ratio between the mean bias in White and Black subjects in the cohort study was 6.6 and 5.47 for light and dark skin, respectively, from the Monte Carlo model. A linear multiplication factor of 1.23 and exponential factor of 1.8 were applied to moderate and dark skin calibration curves, resulting in similar alignment.

**Conclusions:**

This study underpins the careful re-assessment of pulse oximeter designs to minimize bias in ${\mathrm{SpO}}_{2}$ measurements across diverse populations.

## 1 Introduction

Pulse oximeters are non-invasive, dual-wavelength optical devices that operate based on the principles of photoplethysmography (PPG). They are used for the continuous monitoring of arterial oxygenation and are usually placed on the finger, the ear, and the foot (mainly in neonates). The need to assess the performance of pulse oximeters is increasingly gaining attention due to the recent and ongoing concerns of the differential accuracy in individuals with different skin pigmentations.^1^ Evidently, the effect of skin pigmentation on pulse oximetry measurement remains unclear or inconclusive due to a number of reasons. For instance, a common limitation seen in retrospective studies is the lack of consistency in the number of volunteers between different skin types, which primarily arises from the challenging and inconsistent methods for the classification of skin pigmentation.

Over the past few years, there has been continuous research in this field, building upon findings first reported by Sjoding et al.^2^ Their study highlighted a significant disparity in the detection of occult hypoxemia between Black patients and White patients, with Black patients exhibiting nearly three times the frequency of undetected hypoxemia when using pulse oximeters. In the wake of these findings, both pulse oximetry companies and research laboratories have undertaken comprehensive retesting of various over-the-counter and commercial pulse oximeters to assess their accuracy particularly in individuals with darker skin pigmentation.^3^ Although some have shown through their studies that the accuracy of their devices is not affected by melanin,^4^^–^^6^ the majority have yielded a mix of opinions regarding their statistical and clinical significance. Hence, researchers have shifted their focus toward investigating the potential sources of inaccuracy in pulse oximetry, primarily from an engineering hardware perspective, in an effort to unravel the underlying issues.

Currently, there is research to suggest that higher concentrations of melanin, which are often found in individuals with darker skin, could influence the transmission of red light.^7^ This influence could potentially lead to errors in the calculation of the ratio of ratios (R) value, consequently resulting in incorrect oxygen saturation measurements. Rea and Bierman^8^ expanded on the idea by suggesting that the bias associated with skin pigment, inherent in currently available pulse oximetry, could be effectively mitigated when using narrowband light sources. The rationale behind this lies in the fact that LED sources, with their distinct spectral responses, emit a range of wavelengths and not only the peak wavelength of light. This broader spectrum of emitted light can lead to varying interactions with melanin content in the skin, especially at higher concentrations. Furthermore, recent research by Cabanas et al.^9^ continued to support this hypothesis by reinforcing the idea that monochromatic light sources can minimize the positive bias associated with individuals with darker skin pigmentation. Overall, these suggestions align with the notion that, by employing specific wavelengths of light that are less sensitive to changes in skin color, pulse oximeters can offer more accurate and reliable readings for diverse groups of individuals.

In the context of pulse oximetry and the challenges associated with skin pigmentation, computational models utilizing the Monte Carlo (MC) technique are particularly crucial in predicting the outcomes of complex systems, such as biological tissue. Monte Carlo simulations have proven to be a powerful tool in modeling the stochastic behavior of light in biological tissue models. The limited research on the absorption spectra of different melanin concentrations, combined with the standardized calibration algorithms primarily designed and tested on individuals with lighter skin, underscores the need for these types of simulations. Some research has explored how variations in melanin concentration could impact levels of bias (${\mathrm{SpO}}_{2}-{\mathrm{SaO}}_{2}$) or the implications of the differences in important parameters such as the ratio between the alternating current (AC) and direct current (DC) (perfusion index, PI) between light and dark skin.^10^^–^^14^ For instance, Arefin et al.^10^ utilized the Monte Carlo technique to conduct random sampling on various concentrations of melanin, blood, and bilirubin concentrations. These samples represented a cohort that aligns with Food and Drug Administration regulations for pulse oximeter calibration, i.e., for 20% of the participant pool to be darkly pigmented. Their findings showed an overestimation of ${\mathrm{SpO}}_{2}$ in Black subjects by presenting the level of bias, which was seen to decrease when the calibration process involved distributions with a greater enrolment of individuals with higher levels of pigmentation. Furthermore, results observed by Hu et al.^13^ on the wrist in reflectance pulse oximetry and Al-Halawani et al.^14^ on the finger for transmittance and reflectance light–tissue interactions at the two operating wavelengths of pulse oximetry (660 nm and 940 nm) were consistent with each other. Both studies showed that (a) the AC and DC ratios increased with decreasing melanin concentration and (b) the effect of melanin concentration on the red-light PPG characteristics should be paid more attention when predicting blood oxygen saturation. This highlights computationally that melanin concentration may have a similar effect on ${\mathrm{SpO}}_{2}$ measurement in both the wrist and finger for transmittance and reflectance pulse oximeter modes.

Evidently, all studies have contributed to some of the concerns regarding pulse oximeter design or have shed light on potential sources of systematic errors that might lead to the overestimation of oxygen saturation in individuals with darker skin pigmentation. However, they highlight the need for refinement of calibration algorithms for pulse oximeters to accommodate diverse skin pigments. Therefore, the objective of this study is to generate simulated pulse oximetry calibration curves for individuals with light, moderate, and dark skin pigmentation for transmittance pulse oximetry using Monte Carlo modeling on the finger. The novelty in this approach lies in the combined selection of the anatomical site under investigation and the pulse oximeter mode to comprehensively understand bias levels across different skin pigmentation groups using simulated calibration curves. From this, the aims of this study are to (a) provide computational evidence to support the data trends observed in clinical studies, (b) better identify the impact of skin pigmentation on ${\mathrm{SpO}}_{2}$ accuracy, and (c) suggest practical solutions for future improvements in current calibration algorithms.

## 2 Materials and Methods

**Fig. 1:**
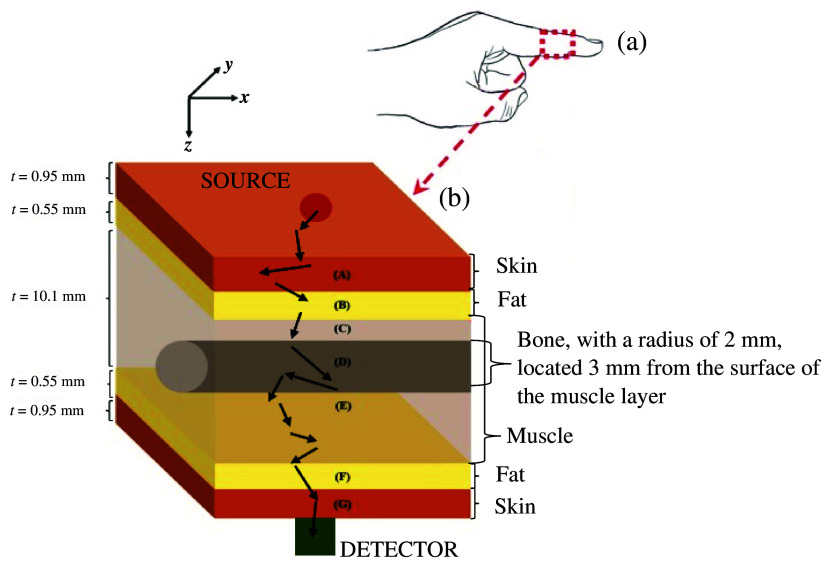
Block diagram of the region of interest under investigation. (a) Human finger (b) Cross section of the part of the finger that is modelled in Monte Carlo simulation. The orange-coloured rectangular slabs (A & G) represent the skin, including the six sublayers. The yellow-coloured rectangular slabs (B & F) represent the fat layers, and the cylindrical bone is located inside the grey rectangular slab, representing muscle. The thicknesses of all the layers are shown on the left of the diagram in units of mm. The source (red circle) and the photodetector (green square) are located on either side of the skin layers, with a source-detector separations equal to the total thickness of the finger of 13 mm. The black arrows show the random path that a photon may traverse before it is detected, due to high levels of scattering.

Previously, Monte Carlo simulations have been employed to investigate the impact of melanin on light–tissue interactions within the visible light spectrum in a monolayer model.^15^ The methodology underpinning these simulations has been adapted for multilayer models of the human finger^16^ to first explore the effect of melanin concentrations on light–tissue interactions.^14^ Using this same finger model, consisting of six skin sublayers, fat, muscle, and bone in alternate order, the same Monte Carlo algorithm is adapted further to investigate the effect of oxygenation and melanin concentration on pulse oximeter calibration curves. The geometry of the region of interest and the sensor configuration are presented in Fig. 1.

The MC model simulated red (660 nm) and infrared (940 nm) light sources to replicate the multiplexing of the two operating wavelengths commonly used in pulse oximeters. The LED sources emitted light based on a Gaussian distribution with a radius of 1 mm. Similarly, the photodetector also had a radius of 1 mm, with an acceptance angle of 90 deg. Central processing unit (CPU) parallelization was utilized in MATLAB to simulate seven arterial oxygen saturation (${\mathrm{SaO}}_{2}$) values ranging between 70% and 100% in 5% increments. To simulate different skin color groups, three distinct skin types were categorized according to the Fitzpatrick scale and characterized by the volume fraction of melanosomes in the epidermis. These were 2.55% for light skin (type I), 15.5% for moderate skin (type IV), and 30.5% for dark skin^17^ and were used to calculate the three absorption coefficients of the epidermis as

$$\mu_{a_{\mathrm{epi}}}({\mathrm{mm}}^{-1})=v_{\mathrm{mel}}\times 6.6\times 10^{10}\times \lambda^{-3.33}+\mu_{a\_w}\times v_{w}+(1-v_{\mathrm{mel}}-v_{w})\times 7.84\times 10^{7}\times \lambda^{-3.255},$$ (1)

where $v_{\mathrm{mel}}$ is the volume fraction of melanosomes in the epidermis, $\mu_{a\_w}$ is the absorption coefficient of water $\left({\mathrm{mm}}^{-1}\right)$,^18^ $v_{w}$ is the volume fraction of water,^19^ and λ is the wavelength of interest (nm).

The diastolic optical properties, blood volumes, and water concentration values for each skin sublayer were defined from previous research (Table 1).^14^ In addition, the systolic phase was simulated by doubling the diastolic blood volume,^25^ which was divided equally between arterial and venous blood.^26^ This assumption was considered viable due to its applicability in previous studies also exploring pulse oximeter modeling and calibration^14^^,^^16^^,^^25^ based on reproducing the conditions of the Schmitt et al. validation study with human pulse oximetry data.^27^ In addition, contributions from venous pulsations have been hypothesized and measured in some studies,^28^^,^^29^ indicating that venous blood may influence PPG signals under certain conditions. Consequently, modeling an equal split allowed for the understanding of the extent of this influence and its implications for ${\mathrm{SaO}}_{2}$ estimation.

**Table 1:** Optical properties and water and blood concentrations for all layers of the finger model for red and infrared light.

Tissue layer/component	$\mu_{a}$ (${\mathrm{mm}}^{-1}$)		$\mu_{s}$ (${\mathrm{mm}}^{-1}$)		g ^20^		$v_{b}$ (%)^19^	v (%)^19^
Wavelength (nm)	660	940	660	940	660	940		
Stratum corneum	0.0495^21^	0.0170^21^	25.62^22^	5.68^22^	0.91	0.94	0	5
Epidermis	-	-					0	20
Light skin	0.7275^21^	0.2297^21^					-	-
Moderate skin	4.2100^21^	1.3023^21^					-	-
Dark skin	8.2438^21^	2.5447^21^					-	-
Papillary dermis	Vary with oxygenation level (70% to 100%)^21^						4	50
Upper blood net dermis							30	60
Reticular dermis							4	60
Deep blood net dermis							10	70
Fat	0.0104^21^^–^^23^	0.0170^21^^–^^23^	6.20^21^^–^^23^	5.42^21^^–^^23^	0.90	0.90	-	-
Muscle	0.0816^21^^–^^23^	0.0401^21^^–^^23^	8.61^21^^–^^23^	5.81^21^^–^^23^	0.88	0.91	-	-
Bone	0.0351^24^	0.0457^24^	34.45^24^	24.70^24^	0.92	0.93	-	-

The absorption coefficients of arterial and venous blood accounted for changes in the arterial and venous oxygenation, which is calculated as

$$\mu_{a_{\mathrm{derm}(n)}}=v_{A(n)}({\text{sat}}_{A}\times \mu_{a_{\mathrm{Hbo}}}+(1-{\text{sat}}_{A})\times \mu_{a_{\mathrm{HHb}}})+v_{V(n)}({\text{sat}}_{V}\times \mu_{a_{\mathrm{Hbo}}}+(1-{\text{sat}}_{V})\times \mu_{a_{\mathrm{HHb}}})+\mu_{a_{w}}\times v_{w(n)}+(1-(v_{A(n)}+v_{V(n)}+v_{w(n)}))\times 7.84\times 10^{7}\times \lambda^{-3.255},$$ (2)

where derm(n) is the n’th sublayer of the dermis: papillary dermis (1), upper blood net dermis (2), reticular dermis (3), and deep blood net dermis (4); ${\text{sat}}_{A}$ is arterial oxygen saturation, ranging between 70% and 100%; ${\text{sat}}_{V}$ is venous oxygen saturation, which is assumed 10% than ${\text{sat}}_{A}$;^30^ $v_{v}$ is the volume fraction of venous blood; $v_{A}$ is the volume fraction of arterial blood; $\mu_{a_{\mathrm{Hbo}}}$ is the absorption coefficient of oxyhemoglobin $\left({\mathrm{mm}}^{-1}\right)$,^31^ and $\mu_{a_{\mathrm{HHb}}}$ is the absorption coefficient of deoxyhemoglobin $\left({\mathrm{mm}}^{-1}\right)$.^31^

To generate calibration curves from the MC model, one million photons were detected to provide transmittance, which is given by the ratio of output to incident intensity. Each photon entering the simulated finger had an initial weighting of 1, which was reduced by subtracting the contribution from surface reflection with refractive indices of 1 and 1.4 for air and tissue, respectively. Incident intensity was calculated by multiplying the incident weight of the photons by the number of photons projected into the finger. This number varied between the three skin types until one million photons were detected, due to varying levels of absorption and scattering, which also varied the computational time. With this data, the ratio of ratios (R) was computed and plotted against their corresponding oxygen saturation levels as

$$R={\left(\frac{I_{d}-I_{s}}{I_{s}}\right)}_{660}/{\left(\frac{I_{d}-I_{s}}{I_{s}}\right)}_{940}=\frac{{\mathrm{ac}}_{660}}{{\mathrm{dc}}_{660}}/\frac{{\mathrm{ac}}_{940}}{{\mathrm{dc}}_{940}},$$ (3)

where $I_{s}$ is the systolic intensity and $I_{d}$ is the diastolic intensity. The numerator shows the normalized transmittance by the red-light source, and the denominator shows the normalized transmittance by the infrared light source.

## 3 Results

**Fig. 2:**
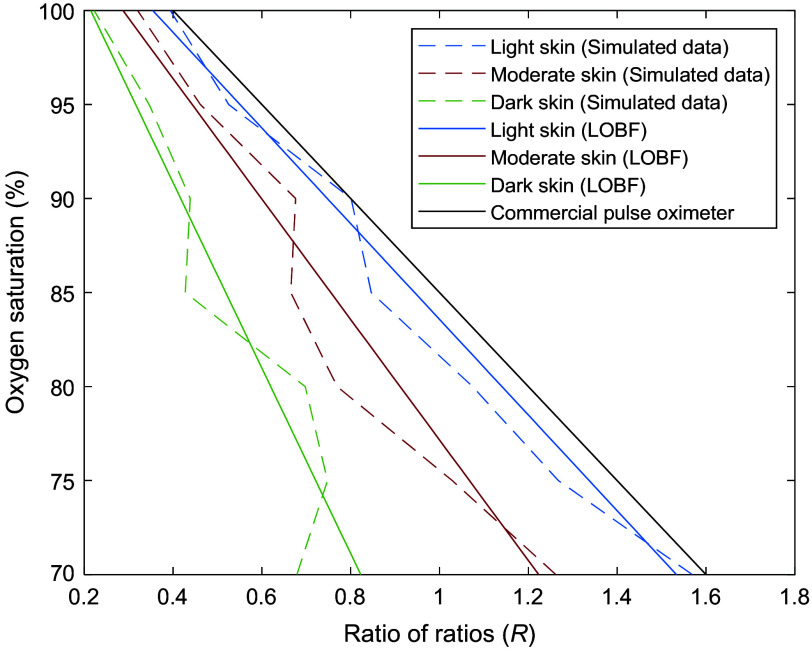
Simulated calibration curves for light, moderate, and dark skin in transmittance mode pulse oximetry. The dashed lines show the computed ratio of ratios using the simulated input and output intensities. The solid lines show the lines of best fit for each calibration curves, compared against a widely used commercial pulse oximeter calibration.

**Fig. 3:**
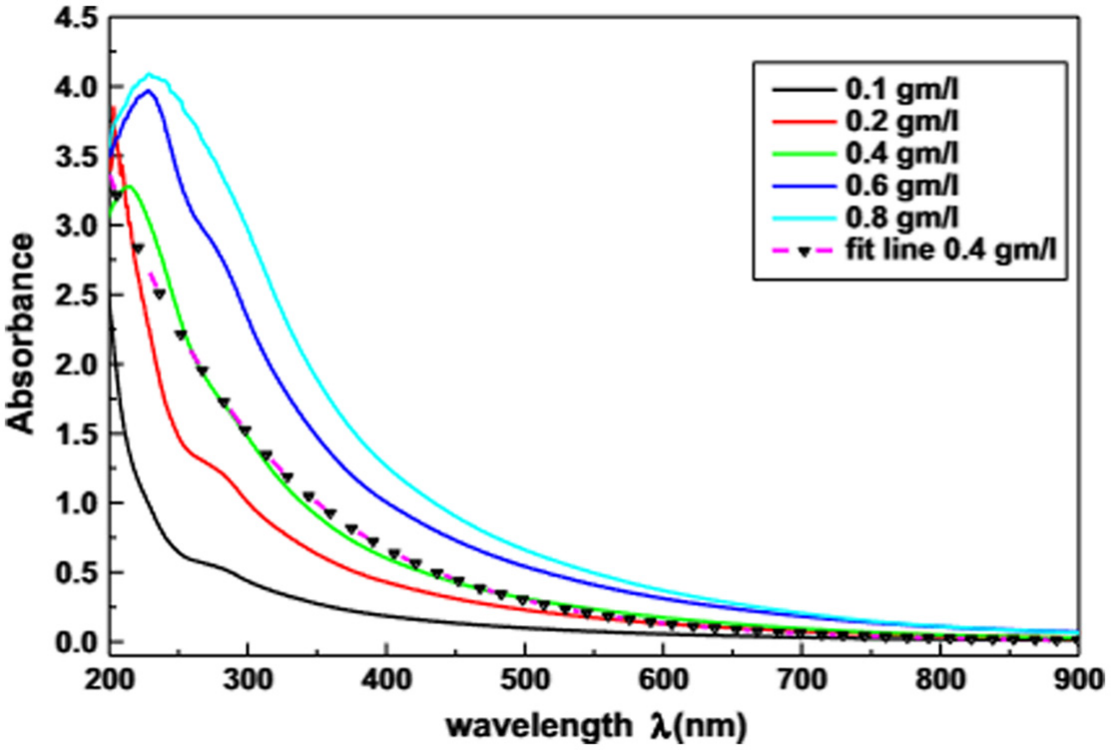
Absorption spectra of different melanin concentrations across the UV-Vis region. Absorbance is given by the ratio between incident and output intensity $I_{in}/I_{out}$ as light passes through the different samples of melanin concentrations. Image produced by Ahmad et al.^32^

Using the data generated from the Monte Carlo simulation, three calibration curves were obtained for light, moderate, and dark skin (Fig. 2). These calibration curves illustrate the correlation between the non-invasive measurement of arterial oxygen saturation and the ratio of ratios. As shown, moderate and dark skin calibration curves are shifted with respect to the simulated calibration curve for light skin. This occurs from a relatively greater increase in absorption of red light as melanin concentration increases for each oxygen saturation level compared with infrared light (Table 2). According to the calculation of the ratio of ratios [Eq. (3)], the value of R decreases if the normalized transmittance in the red wavelength (numerator) decreases and is divided by slight variations in larger normalized transmittance values from the infrared wavelength (denominator). The raw AC/DC values at 660 nm demonstrate a clear trend in which the perfusion index decreases with increasing melanin concentration and oxygen saturation. This trend is consistent with the known absorption characteristics of hemoglobin and melanin. At 660 nm, deoxyhemoglobin absorbs more light, and higher melanin concentrations result in increased absorption, thus reducing the detected signal. On the other hand, the signal at 940 nm appears less influenced by melanin. This is expected because the absorption of light by melanin is significantly lower at this wavelength compared with 660 nm (Fig. 3).^32^ In addition, at a hematocrit level of 45%, oxyhemoglobin and deoxyhemoglobin have similar absorption coefficients at 940 nm ($0.65\;{\mathrm{mm}}^{-1}$ and $0.43\;{\mathrm{mm}}^{-1}$, respectively^31^), in comparison with 0.15 and 1.64 at 660 nm, respectively. Hence, the infrared signal is more stable across different levels of oxygen saturation and melanin concentration. From these findings, it becomes apparent that skin pigmentation influences pulse oximeter calibration algorithms and that a single algorithm may not be suitable for all skin types. This is suggested by the varying behavior of light in the presence of different epidermal melanin concentrations, especially in the visible region.

$$\text{Commercial pulse oximeter }{\mathrm{SpO}}_{2}=110-25R,$$ (4a)

$$\text{Light skin }{\mathrm{SaO}}_{2}=109-25.95R,$$ (4b)

$$\text{Moderate skin }{\mathrm{SaO}}_{2}=109.2-32.69R,$$ (4c)

$$\text{Dark skin }{\mathrm{SaO}}_{2}=110.6-50.31R.$$ (4d)

**Table 2:** Simulated raw AC/DC data at red (660 nm) and infrared (940 nm) wavelengths for all simulated ${\mathrm{SaO}}_{2}$ values (70% to 100%) and skin types.

	Red light (660 nm)						
Skin type	70%	75%	80%	85%	90%	95%	100%
Light	0.4398	0.3845	0.3232	0.2785	0.2631	0.1610	0.1192
Moderate	0.3012	0.2328	0.1759	0.1524	0.2495	0.1383	0.0831
Dark	0.2114	0.1758	0.1607	0.1316	0.1085	0.0811	0.0586
	Infrared light (940 nm)						
Light	0.2748	0.2973	0.2949	0.3227	0.3220	0. 3007	0.2967
Moderate	0.2340	0.2219	0.2240	0.2247	0.3618	0.2937	0.2543
Dark	0.3051	0.2305	0.2258	0.3015	0.2425	0.2296	0.2585

The AC and DC components are calculated using output transmittance during systole and diastole [Eq. (3)].

Following the simulation of the calibration curves for the three skin types, the level of bias was computed between light and dark skin to be compared against the bias observed in the cohort study conducted by Sjoding et al.^2^ In this study, a total of 48,097 paired ${\mathrm{SpO}}_{2}$ and ${\mathrm{SaO}}_{2}$ readings were recorded between two cohorts, each within a 10-min time period. ${\mathrm{SaO}}_{2}$ was directly measured by co-oximetry and included carboxyhemoglobin and methemoglobin saturations.

For direct comparison between the computational and clinical datasets, ${\mathrm{SpO}}_{2}$ was derived for light and dark skin using their respective simulated calibration equations [Eqs. (4b) and (4d)]. Both equations were rearranged to calculate the ratio of ratios for ${\mathrm{SaO}}_{2}$ values between 86% and 92%, as this covered the mean range for ${\mathrm{SpO}}_{2}$ values between 89% and 96% from the cohort study. These R values were then substituted into Eq. (4a), to calculate predicted ${\mathrm{SpO}}_{2}$ based on skin type and then to calculate the difference between true and non-invasive oxygenation. Tables 3 and 4 show ${\mathrm{SaO}}_{2}$ and ${\mathrm{SpO}}_{2}$ data from the clinical and Monte Carlo simulation study for White (light) and Black (dark) subjects, respectively.

**Table 3:** Approximated mean and range of ${\mathrm{SaO}}_{2}$ data for an ${\mathrm{SpO}}_{2}$ range between 89% and 96% ${\mathrm{SpO}}_{2}$ extracted from Sjoding et al.,^2^ who investigated occult hypoxemia in patients identifying as White or Black.

${\mathrm{SpO}}_{2}$ (%)	${\mathrm{SaO}}_{2}$ (%)		Bias (${\mathrm{SpO}}_{2}-{\mathrm{SaO}}_{2}$, %)	
	Mean (range)		Mean (range)	
	White subjects	Black subjects	White subjects	Black subjects
89	88.5 (78 to 99)	86 (83 to 92)	0.5 (11 to −3)	3 (6 to −3)
90	89 (80 to 97.5)	87 (83 to 97)	1 (3 to −7.5)	3 (7 to −7)
91	91 (83 to 98)	87.5 (80 to 97.5)	0 (8 to −7)	3.5 (11 to −6.5)
92	92 (83.5 to 98.5)	89 (82.5 to 96)	0 (8.5 to −6.5)	4 (9.5 to −4)
93	93 (85 to 98)	90 (84 to 97.5)	0 (8 to −5)	3 (7 to −7)
94	93.5 (86 to 98)	91.5 (85 to 97)	0.5 (8 to −4)	2.5 (9 to −3)
95	94 (87 to 98.5)	92 (85.5 to 97)	1 (8 to −3.5)	3 (9.5 to −2)
96	94.5 (89 to 99)	92 (84 to 98)	1.5 (7 to −3)	4 (12 to −2)
			**Mean bias (%)**	
			**0.5 (7.7** to **−4.9)**	**3.3 (8.9** to **−4.3)**
			**Ratio between mean biases**	
			**6.6**	

Bias is calculated by subtracting ${\mathrm{SaO}}_{2}$ from ${\mathrm{SpO}}_{2}$, and the mean bias is calculated to provide a quantitative measure of the ratio of ratios between bias levels in White and Black subjects.

**Table 4:** Simulated oxygen saturation (${\mathrm{SpO}}_{2}$) for light and dark skin.

	Simulated ${\mathrm{SpO}}_{2}$ (output), %		Bias (${\mathrm{SpO}}_{2}-{\mathrm{SaO}}_{2}$), %	
True ${\mathrm{SaO}}_{2}$ (input), %	Light skin	Dark skin	Light skin	Dark skin
86	87.8	96.8	1.8	10.8
87	88.8	97.3	1.8	10.3
88	89.8	97.8	1.8	9.8
89	90.7	98.3	1.7	9.3
90	91.7	98.8	1.7	8.8
91	92.7	99.2	1.7	8.3
92	93.6	99.8	1.6	7.8
			**Mean bias (%)**	
			**1.7**	**9.3**
			**Ratio between mean biases**	
			**5.47**	

The computed ratio of ratios for an ${\mathrm{SaO}}_{2}$ range between 86% and 92% are inputted in the commercial pulse oximeter equation to calculate predicted ${\mathrm{SpO}}_{2}$ with current in-built algorithms. Bias is calculated for both skin types and used to determine the mean ratio between the biases.

The larger bias observed in the Monte Carlo simulation results compared with the clinical study data can be attributed to the relative simplification and assumptions about tissue optical properties and physiological conditions. This may not fully capture the complexities and variability inherent in human subjects, especially those with darker skin pigmentation, which can average out biases. Despite these differences, the similarity in the ratio between the two sets of data (6.6 for the clinical study and 5.47 for the simulation study) suggests that the underlying trends and relationships are consistent across both methods, providing confidence in the overall validity of the simulation approach.

**Fig. 4:**
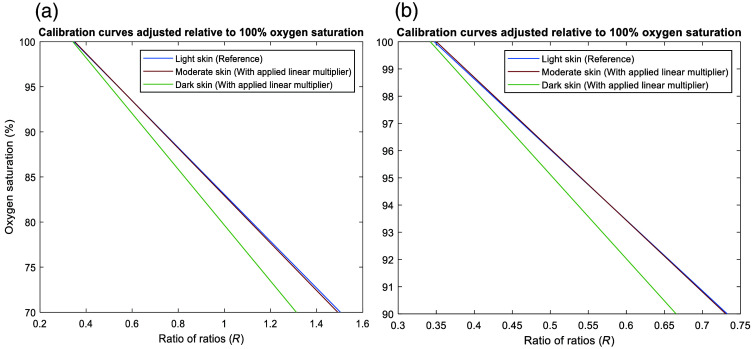
Adjusted calibration curves for moderate and dark skin with an applied linear multiplier relative to light skin at 100% oxygen saturation. (a) Adjusted calibration curves between 70% and 100% oxygen saturation. (b) Adjusted calibration curves between 90% and 100% oxygen saturation (range of interest). The data suggests that the dark skin calibration curve requires a non-linear re-adjustment.

**Fig. 5:**
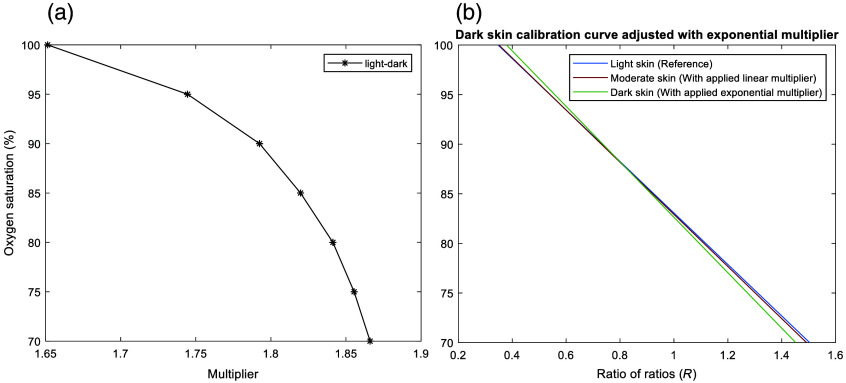
Re-adjustment of the simulated calibration curve for dark skin. (a) Multiplication values for the ratio of ratios between light and dark skin across an oxygenation saturation range of 70% and 100%. (b) Adjusted dark skin calibration curve using the mean of the exponential multipliers. All simulated calibration curves now follow very similar algorithms, suggested by the integration of correctives measures.

Moreover, the feasibility of data manipulation to devise potential solutions for pulse oximeter calibration for moderate and dark skin pigmentation was explored. Given that current pulse oximeters exhibit minimal bias in light skin, this indicates that algorithms may be predominantly tailored to this demographic. Therefore, the light skin calibration curve served as a comparative metric for the calculation of the ratios between the R values of light and moderate skin and light and dark skin at a reference ${\mathrm{SpO}}_{2}$ of 100% (healthy subjects). These calculations yielded multipliers of 1.23 and 1.65, respectively. After initially applying these values across the total oxygen saturation range, it became visually apparent that moderate skin closely aligned with the light skin calibration curve, but not for dark skin below 100% (Fig. 4). This observation suggested that a linear multiplier alone may not suffice for implementing an inherent correction in pulse oximeters for dark skin. Even at an oxygen saturation level of 90% [Fig. 4(b)], a bias of 2.1% was recorded relative to light and moderate skin. Consequently, the ratios between the R values for light and dark skin were recalculated for the entire oxygen saturation range, as depicted in Fig. 5(a). Notably, these ratios suggested an exponential trend, which can be attributed to the decrease in the pathlength and photon penetration depth due to increased absorption (characterized by a higher absorption coefficient value), thus reducing the perfusion index, especially at higher oxygen saturation levels in the dermal layers [Eq. (2)]. The mean of these multipliers was equal to ∼1.8, which was then applied to the original dark skin calibration curve, resulting in significant adjustments that brought all calibration algorithms for the three skin types into close proximity [Fig. 5(b)]. Hence, the adjustments suggest the feasibility of integrating corrective measures into pulse oximeter designs to safeguard ${\mathrm{SpO}}_{2}$ accuracy against the influence of skin pigmentation.

## 4 Discussion

The discrepancies observed in medical devices such as pulse oximeters have undoubtedly impacted clinical outcomes, particularly over the past 5 years. This arises from inconsistencies in data collection and poor data quality from in vivo calibration experiments, which have led to underrepresented datasets and occurrences of physical and computational biases in hardware and software. With this in consideration, there is also a lack of adequate training provided to clinicians to recognize inequity issues when monitoring patients’ oxygen saturation levels during and after hospital admission or in providing guidance to patients in the use of pulse oximeters at home. This ongoing concern has called for an independent review initiated by the UK Secretary of State for Health and Social Care to consult a group of healthcare professionals dedicated to achieving equity in healthcare.^33^ They proposed several recommendations, one of which states

“Innovators, researchers and manufacturers should cooperate with public and patient participants to design better, smarter oximeters using innovative technologies to produce devices that are not biased by skin tone. This could include developing enhanced algorithms for oximeter device software to address measurement bias”.

Aligned with this recommendation, the data generated from the current Monte Carlo model has successfully simulated calibration curves with light, moderate, and dark skin, suggesting that a single pulse oximeter algorithm may not be universally acceptable for all skin types. Evidently, different interactions of light in darker epidermis cause the calibration curves to shift with respect to the light skin calibration curves, resulting from greater absorption exhibited by higher concentrations of melanin in the visible spectrum.

Moreover, the results from the MC simulation were validated against experimental data to ensure consistency with observations of oxygen saturation variations relative to skin pigmentation in clinical settings. The ratio of the mean biases between White and Black subjects from the cohort study approximately matched the ratio from the simulation study between light and dark skin mean biases, with a difference of 1.13%. Consequently, further analysis explored the feasibility of adjusting pulse oximeter calibration across diverse skin types to suggest potential solutions for measurement bias. It was seen that the simulated dark skin calibration curve was only fully adjusted with respect to the light skin curve after applying an exponential multiplication factor, in comparison to the linear multiplication factor applied to the moderate skin calibration curve. This indicated that, by integrating different corrective measures into pulse oximeter designs, the influence of skin pigmentation on ${\mathrm{SpO}}_{2}$ accuracy can be minimized. Overall, such calibration adjustments in pulse oximeters can improve accuracy and reliability for enhanced patient care and monitoring in various healthcare and at-home settings. However, future work on the effect of skin pigmentation on reflectance pulse oximeters also requires investigation, particularly due to the rise and accessibility of consumer wearables.

## 5 Limitations

Despite the robust findings of this study, several limitations should be noted. First, the variability in output intensity values at the two wavelengths during systole and diastole should be considered by running the model multiple times to ensure data repeatability and/or reduce the deviation of the data from the lines of best fits. Achieving a balance between computational time and model accuracy posed challenges, particularly when simulating dark skin. Ideally, with greater computational speed and power, simulations would be conducted for more than one million detected photons to minimize randomness, which may result in an increased level of noise. In addition, the assumption that systolic blood volume is twice the diastolic blood volume and is equally split between arterial and venous compartments could be refined. A more representative pressure–volume relationship and physiological activities related to the cardiac cycle can be incorporated in the future, which may impact the optical coefficients of arterial and venous blood and therefore the dermal layers. Furthermore, although melanin concentration was assumed to be uniform on both sides of the finger, this simplification may not reflect reality. Finally, there is a lack of research on melanin distribution across different skin color groups due to the effect of environmental factors such as ultraviolet (UV) radiation. However, future models could benefit from simulating randomized melanin concentration levels on the first and last epidermal layers to evaluate this effect on the calibration curves. Overall, addressing these limitations in future studies would enhance the model’s accuracy in reflecting a more realistic modeling of the human finger.

## 6 Conclusions

This study has intended to contribute to the scientific knowledge associated with pigmentation-related bias in pulse oximeters through a Monte Carlo simulation. The simulated results have provided quantitative evidence to rectify the shift occurring in moderate and darker skin pigmentations, which, moving forward, can be considered by pulse oximeter manufacturers. Furthermore, the MC model has shown that melanin concentration, as the primary determinant of skin color, is suitable for the computational representation of different skin types. Hence, the retesting of pulse oximeters with additional in-built algorithm adjustments, as well as more precise in vitro and in vivo investigations, is encouraged.

## Data Availability

The code and some of the data that support the findings of this article are not publicly available due to privacy. Other data not included in this article can be requested from the author at Raghda.al-halawani@city.ac.uk.
